# Genetic Variants of the *ATIC* Gene and Therapeutic Response to Methotrexate in Patients with Rheumatoid Arthritis

**DOI:** 10.3390/ijms26094013

**Published:** 2025-04-24

**Authors:** Sergio Gabriel Gallardo-Moya, Laura Gonzalez-Lopez, Betsabe Contreras-Haro, Mario Alberto Mireles-Ramirez, Alejandra Villagomez-Vega, María Cristina Morán-Moguel, Miriam Méndez-Del Villar, María Luisa Vazquez-Villegas, Jorge Ivan Gamez-Nava, Ana Miriam Saldaña-Cruz

**Affiliations:** 1 Doctorado en Farmacología, Departamento de Fisiología, Centro Universitario de Ciencias de la Salud (CUCS), Universidad de Guadalajara, Guadalajara 44340, Jalisco, Mexico; 2 Departamento de Fisiología, Instituto de Terapéutica Experimental y Clínica, Centro Universitario de Ciencias de la Salud (CUCS), Universidad de Guadalajara, Guadalajara 44340, Jalisco, Mexicoivangamezacademicoudg@gmail.com (J.I.G.-N.); 3 Departamento de Ciencias Biomédicas, Centro Universitario de Tonalá (CUT), Universidad de Guadalajara, Tonalá 45425, Jalisco, Mexicoalejandra.villagomez5427@academicos.udg.mx (A.V.-V.); miriam.mendez@academicos.udg.mx (M.M.-D.V.); 4 Unidad de Investigación Biomédica 02, Hospital de Especialidades Centro Médico Nacional de Occidente, IMSS, Guadalajara 44329, Jalisco, Mexico; 5 Dirección de Investigación y Educación, Hospital de Especialidades Centro Médico Nacional de Occidente, IMSS, Guadalajara 44329, Jalisco, Mexico; 6 Departamento de Disciplinas Filosófico, Metodológicas e Instrumentales, Centro Universitario de Ciencias de la Salud (CUCS), Guadalajara 44340, Jalisco, Mexico; cristina.moran@academicos.udg.mx; 7 Instituto Regional de Investigación en Salud Pública, Programa de Doctorado y Coordinación del Programa de Doctorado en Salud Pública, Ciencias de la Salud Pública, Centro Universitario de Ciencias de la Salud (CUCS), Universidad de Guadalajara, Guadalajara 44340, Jalisco, Mexico; luisa.vazquez@academicos.udg.mx

**Keywords:** *ATIC* gene, AICAR transformylase, methotrexate, therapeutic response, rheumatoid arthritis

## Abstract

Methotrexate (MTX) is the conventional synthetic disease-modifying anti-rheumatic drug (csDMARD) recommended as the first-choice anti-rheumatic drug for rheumatoid arthritis (RA). However, responses to MTX may be influenced by genetic variants. We aim to evaluate the association of the rs2372536, rs4673990, and rs4673993 genetic variants of the *ATIC* gene with therapeutic failure of MTX in patients with RA. A case–control study was performed. Disease activity was measured using the disease activity score based on erythrocyte sedimentation rate (DAS28-ESR). RA patients were classified into two groups: (a) responders (DAS28-ESR ≤ 3.2), which is the group of patients who did respond to methotrexate, and (b) non-responders (DAS28-ESR > 3.2), which is the group of patients who did not respond to methotrexate. Serum levels of the 5-aminoimidazole-4-carboxamide ribonucleotide (AICAR) enzyme and Interleukin-6 (IL-6) were quantified using an enzyme-linked immunosorbent assay (ELISA). Genotyping of *ATIC* genetic variants was performed with quantitative polymerase chain reaction (qPCR) using TaqMan probes. A total of 260 patients with RA were included. In total, 142 (54.6%) were non-responders to MTX. IL-6 levels were increased in the non-responder group (*p* = 0.002), while no statistical differences were observed in the AICAR levels. The variables associated with non-response were higher HAQ-Di, weekly MTX dose, glucocorticoid use, erythrocyte sedimentation rate, and carriers of the polymorphic homozygous variant of rs4673993 (OR = 4.5, 95% CI: 1.04–19.34; *p* = 0.04). The use of sulfazaline offered protective effects. Our findings indicate that the polymorphism rs4673993 gene variant of the AICAR protein may significantly influence MTX resistance. Therefore, these results support the importance of the pathway generating extracellular adenosine and its effects on promoting the immune regulation for the mechanism of MTX therapy of RA.

## 1. Introduction

Rheumatoid arthritis (RA) is an autoimmune, systemic, and inflammatory disease that affects the synovial joints, causing chronic pain, bone erosions, and functional disability [1]. The prevalence estimated in the Mexican population varies between 0.5% and 1.6% [2,3]. Early treatment with conventional synthetic disease-modifying anti-rheumatic drugs (csDMARDs) is essential to controlling disease activity and reducing its progression, where methotrexate (MTX) is the csDMARD recommended as the first-choice anti-rheumatic drug for RA [4]. MTX at low doses has considerably changed the therapeutic approach and outcomes of RA, as many patients do not require additional treatments [5]. However, responses to MTX vary, with non-response rates between 46% and 54% [6,7]. Even patients who have had an adequate response to MTX are treated with glucocorticoids (GCs) as a bridging therapy, combined with other csDMARDs and biological disease-modifying anti-rheumatic drugs (bDMARDs), to achieve better treatment response rates [4]. The use and efficacy of MTX in RA treatment will be enhanced by a better understanding of the pharmacology and anti-inflammatory mechanisms of action of this drug.

MTX diffusion through cell membranes is taken up through reduced folate transporter 1 (RFC1), which is polyglutamated by folylpolyglutamate synthase (FPGS) to have a higher affinity for enzymes of the folate pathway. The active metabolite of MTX, polyglutamate, accumulates intracellularly and inhibits 5-aminoimidazole-4-carboxamide ribonucleotide (AICAR) transformylase, which generates intracellular accumulation of AICAR and has anti-inflammatory and immune effects [8]. Response variation to treatment may be influenced by several factors, including alterations in drug metabolism genes. Genetic variants may modify gene expression and, thus, modulate the interaction protein expression with the drug, making the response inefficient.

The *ATIC* gene encodes the AICAR protein, and it is located on chromosome 2 in region 35 of the long arm and contains 17 exons and 16 introns [9]. Recently, single-nucleotide polymorphisms (SNPs) that could be implicated in structural changes in this protein have been characterized [10]. The rs2372536 gene variant with a C>G change is found in exon five and generates a substitution of threonine for serine at position 116 of the *ATIC* gene [11]. The rs4673990 gene variant with an A>G change in intron 12 and the rs4673993 gene variant with a T>C change in intron 13 are implicated in linkage disequilibrium with other SNPs that change protein structure or alter gene expression [12].

Therefore, this study aims to evaluate the association of the rs2372536, rs4673990, and rs4673993 gene variants of the *ATIC* gene with therapeutic failure of MTX in patients with RA.

## 2. Results

Table 1 describes the clinical characteristics, genotypes, and allele frequencies for the three rs *ATIC* genes of the 260 patients with RA included in this study. In terms of clinical characteristics, these patients had a mean age of 57 ± 12 years; 95% of the RA patients were women, and they had a mean disease duration of 13 ± 9.3 years and a mean HAQ-Di of 0.52 ± 0.53. All patients were treated with DMARDs; 86 (33%) patients were treated with monotherapy with MTX with a mean dose of 13.2 ± 6.3 mg per week, whereas 174 (67%) patients had a combined treatment with two DMARDs. Of the patients, 42% consumed glucocorticoids, with a mean dose of 3.28 ± 3.9 mg. In relation to the *ATIC* gene genotypes of the gene variants rs2372536, rs4673990, and rs4673993, the homozygous wild-type genotype was found in 34% (CC), 33% (CC), and 42% (CC) of patients, the heterozygous genotype was found in 48% (CG), 49% (AG), and 47% (TC) of patients, and 18% (GG), 18% (AA), and 11% (TT) of patients presented the polymorphic homozygous genotype.

Table 2 compares RA patients with DAS28 ≤ 3.2 vs. RA patients with DAS28 > 3.2. Non-responders (DAS28-ESR > 3.2) had a lower frequency of alcoholism (*p* < 0.04), a higher functional disability score (*p* < 0.01), and higher ESR, CRP (*p* < 0.01 and *p* < 0.05, respectively), and IL-6 levels (*p* < 0.002) in comparison to responders. In addition, non-responders had a higher weekly MTX dose (*p* < 0.05), higher use of glucocorticoids and sulfasalazine (*p* < 0.05 in both cases), and a higher glucocorticoid dose (*p* < 0.01). We did not observe differences in other variables, including gender and serum AICAR levels between responders vs. non-responders.

Table 3 compares the genotypic and allele frequencies of rs2372536, rs4673990, and rs4673993 of the *ATIC* gene between responders (DAS28 ≤ 3.2) and non-responders (DAS28 > 3.2). We also inferred the haplotypes with eight possible combinations. The most frequent were (1) CAC (40%), (2) GGT (32%), and (3) GCG (15%). No differences were observed in the genotypes of the genetic variants analyzed, the genetic models, and the haplotypes between responders and non-responders.

Table 4 presents the results of univariate and multivariate logistic regression analysis to identify risk factors for therapeutic non-response to MTX in RA patients. In the multivariate analysis, HAQ-Di (OR = 3.92, 95% CI: 2.02–7.6; *p* < 0.01), weekly MTX dose (OR = 1.06, 95% CI: 1.01–1.14; *p* = 0.46), glucocorticoid use (OR = 2.87, CI95%: 1.44–5.73; *p* < 0.01), ESR (OR = 1.08, CI95%: 1.04–1.12; *p* < 0.001), and the presence of the polymorphic genotype of the rs4673993 variant (OR = 4.5, 95% CI: 1.04–19.34; *p* = 0.04) increased the risk of therapeutic non-response. On the other hand, the use of sulfazaline (OR = 0.49, 95% CI: 0.25–0.95; *p* = 0.03) offered protective effects.

Table 5 shows the sub-analysis of RA patients with MTX as monotherapy. Patients with non-response had a higher functional disability score (*p* < 0.01), a higher ESR (*p* = 0.01), and higher AICAR levels (*p* = 0.04) in comparison to responder patients on methotrexate monotherapy.

## 3. Discussion

The present study analyzes the potential association of the gene variants rs2372536, rs4673990, and rs4673993 of the *ATIC* gene and the therapeutic failure of MTX in RA. The variants rs2372536 and rs4673990 were not associated with therapeutic failure in Mexican patients with RA. In this study of Mexican–Mestizo patients with RA, we identified prevalence of the heterozygote in the three variants that were the most frequently observed in the Mexican–Mestizo population with RA, similar to the results for other populations [13].

These results disagree with the results obtained by two separate studies, those of Ankita et al. and Kurkazawski, who described an association between the GG genotype in the *ATIC* (rs2372536) polymorphism and the response to MTX therapy [14,15]. The differences may be because MTX was used as monotherapy in these studies. However, in current medical practice, most of the time, combined therapy is prescribed because a number of studies point out that a low proportion of patients in remission is produced when MTX is used as monotherapy [16]. On the other hand, the results of the studies are contradictory, with a meta-analysis showing an association between GG + GC genotypes and non-response to MTX therapy (OR = 1.572, 95% CI 1.146–2.156, *p* = 0.005) [17].

Regarding the rs4673993 gene variant, several studies have reported an association between this gene variant and an increased remission rate in RA patients’ treatment with MTX [17]. Additionally, a meta-analysis conducted by Chen reported that this polymorphism had an association with responsiveness in the recessive model (OR: 2.54; 95% CI: 1.23–5.26) [18]. Sha HX et al. reported that both gene variants (rs4673993 and rs2372536) can be in linkage disequilibrium [19]. Our results did not show linkage disequilibrium between the markers analyzed, and no association was found between haplotypes and treatment failure.

Abdallah et al. reported an association with non-response to MTX in the rs4673990 variant in Egyptian patients [20]. In addition, 26 polymorphisms were studied to evaluate the influence of MTX therapeutic outcomes in Portuguese RA patients; from these, eight genotypes were found to be possible predictors of MTX non-response, among which were *ATIC* rs2372536 C carriers and *ATIC* rs4673993 T carriers. In this study, other phenotypes, such as toxicity, were additionally associated with these genotypes [7].

In our study, we observed that rs4673993, in the univariate analysis, confers an association to resistance to MTX. This finding has not been published in any other study.

However, the observed results are not consistent with other studies due to many reasons, such as diversity in the analyzed populations, variations in the MTX response criteria, and different sample sizes of the groups under study.

To the best of our knowledge, we have reported the first allelic and genotypic frequencies in Latin American countries for the three *ATIC* variants in therapeutic response to MTX in patients with RA. The haplotypes of these three variants are also described for the first time, as well as their frequencies in the Mexican population.

Methotrexate polyglutamate is known to inhibit AICAR in its mechanism of action, resulting in intracellular accumulation of AICAR and increased release of adenosine; adenosine binds to cell surface receptors and suppresses many inflammatory and immune reactions. However, the gene variants implicated in this study may be involved in the imbalance in the mechanism of action of MTX. rs2372536 is located within exon 5 of the gene and generates a substitution of threonine for serine at position 116 in the *ATIC* gene, causing reorganization of the protein structure in the N-cap, and therefore, it affects substrate binding and AICAR enzyme activity, causing a phenotypic change in response to MTX. In contrast, intronic gene variants, such as *ATIC* rs4673993 and rs 4673990, could decrease enzyme activity by being in linkage disequilibrium with other gene variants that change protein structure or transcriptional regulation of the enzyme.

Therapeutic response is a multifactorial process involving multiple factors, including disease-specific, individual, and genetic factors. In this study, the prevalence of non-responders to therapy with MTX was 54.6%; this result is similar to those reported by Strand (46%) and Lima (56%) in a Caucasian population. Other studies have described higher response rates in MTX monotherapy, with Soukup reporting 70.4% and Kobold reporting 80.7% due to differences in patient characteristics and cut-off points for defining a response [6,7].

In the treatment with MTX for rheumatic diseases such as RA, the active ingredient, methotrexate polyglutamate, potently inhibits ATIC via intracellular accumulation [8]. AICAR accumulates intracellularly in the tissues of methotrexate-treated mice. AICAR inhibits AMP deaminase and adenosine deaminase, which drives adenine nucleotides into the extracellular space; these adenine nucleotides are converted to adenosine by the action of the cell surface enzymes ectonucleoside triphosphate dephosphorylase 1 (CD39) and ecto-5′-nucleotidase (CD73). Adenosine is a potent stimulant for the A1a, A2a, A2b, and A3 family of adenosine receptors, which have inhibitory effects on inflammatory cells. Thus, adenosine has a vital role in mediating anti-inflammatory effects. Adenosine that is released diminishes the adherence of neutrophils and inhibits the function of natural killer cells, monocytes/macrophages, and T-lymphocytes, thus producing potent anti-inflammatory effects [9].

Our study found no differences in the serum AICAR levels between responders and non-responders. The AICAR protein levels are mainly expressed in intracellular space, and a small percentage are expressed in extracellular space [21,22].

The three variants of the *ATIC* gene analyzed in the present study were selected for their possible biological significance and include one missense variant (rs2372536) and two intronic variants (rs4673990 and rs4673993) involved in altering the structure and function of the encoded protein; however, other variants of the ATIC gene, which may be associated with the response to treatment with MTX in patients with RA, should also be analyzed, even at the level of protein expression, using another methodology such as flow cytometry.

AICAR, being an AMP kinase (AMPK) analog, is involved in other signaling pathways at the extracellular level due to the receptors involved in these pathways. As a cell-permeable nucleotide, AICAR enters cells through adenosine transporters and is phosphorylated by adenosine kinase at the AICAR. AICAR activates AMPK but is 40 to 50 times less potent than adenosine monophosphate (AMP) in activating AMPK and accumulates in high concentrations in the cytoplasm; thus, it is likely that AICAR may have several AMPK-independent effects [22].

As for future perspectives, due to a lack of studies or conflicting studies analyzing the impact of the SNPs in protein function and/or MTX therapeutic outcome, further evidence is necessary to support the interpretation of our results. Since we could not genotype all the selected SNPs because of technical limitations of the genotyping technique, other techniques should be considered due to the possible importance of SNPs and due to the lack of studies analyzing the SNPs that were excluded. Further studies should consider that a possible synergistic effect between MTX and other DMARDs could influence the associations between genetic polymorphisms and MTX therapeutic outcome. Finally, in the near future, algorithms combining pharmacogenetics and clinical data should be developed using these and other genetic association study results after proper validation in order to aid physicians in therapeutic choices at diagnosis.

Hence, composite score analysis comprising clinical and genetic parameters should be considered in the search for a robust marker for drug efficacy.

This study has some limitations derived from the case–control design in which patients were classified by disease activity; patients were not followed up to identify changes in disease activity and any need for changes in the therapeutic approach. Additionally, this study did not evaluate other clinical parameters that may be associated with therapeutic failure of MTX. Longitudinal studies are needed to corroborate the information identified within this study.

This study is one of the first in Latin America to evaluate the impact of genetic variants on the therapeutic response in patients with rheumatoid arthritis. Additionally, this work relates serum parameters with *AICAR* gene polymorphisms of high prevalence in the Mexican population. In addition, the clinical response of patients to MTX treatment, which is the mainstay of management in most patients with RA, both as monotherapy and combined therapy, is evaluated.

## 4. Materials and Methods

A case–control study was used.

### 4.1. Study Population

In total, 260 patients with RA were included. Patients were self-identified as Mexican–Mestizo (defined as individuals who were born in Mexico and were re descendants of the original autochthonous inhabitants of the region and of individuals who were mainly Spaniards, with three generations of Mexican ascendants). Patients were recruited from October 2021 to December 2022 at the Institute of Experimental and Clinical Therapeutics (INTEC) of the University Center of Health Sciences (CUCS). They were referred from a second-level hospital in the rheumatology area (Regional General Hospital 110 of the Mexican Institute of Social Security, IMSS) in Guadalajara, Mexico. Inclusion criteria included an age > 18 years old, meeting the 1987 American College of Rheumatology criteria for RA diagnosis [23], being treated with MTX monotherapy or MTX combination therapy with csDMARDs or bDMARDs for at least 3 months, and providing voluntary informed consent prior to participation in this study. Patients with overlap syndrome (features of two autoimmune connective tissue diseases present in the same patient) and acute or chronic active infections (including hepatitis B or C, human immunodeficiency virus, or tuberculosis) were excluded. The Research Committee for Ethics and Biosafety of the University of Guadalajara approved this study (approval number: Cl-05021), considering ethical, legal, and regulatory standards and norms for research involving human subjects, the principles of the 75th Declaration of Helsinki (last revision: Helsinki, Finland, October 2024), and the national norms for research studies in humans.

### 4.2. Clinical Setting

Qualified investigators and clinicians evaluated patients. Clinical and demographic characteristics were recorded using a structured questionnaire. The clinical features of RA included disease activity evaluated by a 28-joint score (DAS28). DAS28 includes the quantification of 28 swollen joints and 28 painful joints, an index based on a patient-perceived visual analog scale, and erythrocyte sedimentation rate (ESR) as an acute phase reactant (DAS28-ESR) [24]. Moreover, for patients’ physical function, the Health Assessment Questionnaire Disability Index (HAQ-Di), which assessed the amount of difficulty in performing activities of daily living, was analyzed. A cut-off point > 0.6 was defined as functional disability [25]. Body mass index (BMI) was measured in weight (kilograms)/height (meters^2^). Additionally, a peripheral blood sample was obtained, after 8 h of fasting, by venipuncture.

### 4.3. Therapeutic Response to Methotrexate Assessment

The RA patients were classified into two groups according to DAS28-ESR score: (a) responders to csDMARDs, a low-activity group with DAS28-ESR ≤ 3.2(control), and (b) non-responders to csDMARDs, a high-activity group with DAS28-ESR > 3.2 (cases).

### 4.4. Laboratory Measurements

Levels of rheumatoid factor (RF) (IU/mL) and C-reactive protein (CRP) (mg/mL) were quantified using nephelometry. Erythrocyte sedimentation rate blood tests (mm/h) were performed using the Wintrobe method.

Serum levels of the AICAR enzyme and Interleukin-6 (IL-6) were quantified. Quantification of AICAR enzyme levels was determined by an enzyme-linked immunosorbent assay (ELISA). In a commercial kit (MyBioSource, San Diego, CA, USA), Human AICAR Immunoassay), the AICAR enzyme was detected in the 12.5–800 pg/mL range, with a sensitivity of 7.5 pg/mL and an interassay precision of <10%. For IL-6 levels, a commercial kit (R&D Systems, Minneapolis, MN, USA) Quantikine Human IL-6 Immunoassay was used, and levels were detected in the range of 3.13–300 pg/mL, with a sensitivity of 0.70 pg/mL and an interassay precision of 4.2%.

### 4.5. ATIC Genotypes

For genomic purposes, whole-blood samples were collected and harvested in vacutainer tubes containing ethylenediaminetetraacetic acid as an anticoagulant. Genomic DNA was obtained from blood samples using the modified Miller technique [26] and quantified using a NanoDrop spectrophotometer. DNA was diluted in Tris–EDTA buffer to 20 ng/μL and placed in 200 μL propylene cryotubes (Eppendorf™, Hamburg, Germany). Genotyping of *ATIC* rs2372536, rs4673990, and rs4673993 gene variants was performed by quantitative polymerase chain reaction (qPCR) using TaqMan probes (Waltham, MA, USA). The TaqMan Assay IDs were C__16218146_10, C__28017839_30, and C__362264_10; the StepOne™ Real-Time Polymerase Chain Reaction (qPCR) System was employed for this purpose, and it was used according to the manufacturer’s protocol (Applied Biosystems, Waltham, MA, USA). All results were independently verified by two investigators blinded to patient information. In the case of ambiguous results, the sample was analyzed a second time.

The eight possible haplotypes for three biallelic SNPs were inferred considering the following allele order (from centromere to telomere): rs2372536 (allele 1 = C, allele 2 = G), rs4673990 (allele 1 = A, allele 2 = G), rs4673993 (allele 1 = C, allele 2 = T). Thus, the three most frequent haplotypes were (1) CAC, (2) GGT, and (3) GCG.

### 4.6. Statistical Analysis

Quantitative variables were expressed as means ± standard deviations (SDs), and qualitative variables were expressed as frequencies and percentages (%). Comparisons of proportions between groups were computed using the chi-square test and between means using the independent sample *t*-test. Multivariate analysis was performed to analyze factors associated with resistance to MTX. Covariates were variables with a *p*-value ≤ 0.20 in univariate analyses or those with biological plausibility for association to resistance to MTX. Adjusted odds ratios (ORs) and their 95% confidence intervals (95% CIs) were obtained in these models using the backward method.

The following genetic models were adopted in the present study: dominant (CC vs. CG + GG) and recessive (CG + CC vs. GG) for rs2372536, dominant (AA vs. AG + GG) and recessive (AG + AA vs. GG) for rs4673990, and dominant (TT vs. TC + CC) and recessive (TC + TT vs. CC) for rs4673993. The Hardy–Weinberg equilibrium (HWE) in the control subjects was determined by comparing the observed and expected data using the chi-square test.

A *p*-value was considered significant if *p* ≤ 0.05. Odds ratios and their confidence intervals were obtained using EPI-INFO version 7.2 (Epi Info TM, Atlanta, GA, USA). Data were analyzed using SPSS software 23.0 (SPSS Inc., Chicago, IL, USA). Arlequin software version 2007 (University of Bern, Switzerland) was used to infer haplotypes and assess the Hardy–Weinberg equilibrium.

A sub-analysis was performed with the patients who only had MTX monotherapy divided into two groups—responder DAS28-ESR ≤ 3.2 vs. non-responder DAS28-ESR > 3.2—and the main variables of interest.

## 5. Conclusions

This study concludes that serum AICAR levels were significantly related to MTX resistance in a subgroup of patients and that this is due to the polymorphism within the rs4673993 gene variant of the AICAR protein. The mutation is likely to limit the release of adenosine from MTX-treated cells. These results support the importance of the MTX-sensitive pathway that generates extracellular adenosine, which can promote immune regulation as therapy for RA. No association was observed for variants rs2372536 and rs4673990. Our study supports the need for further investigation on the MTX-sensitive pathways that generate extracellular adenosine for therapy for inflammatory diseases.

## Figures and Tables

**Table 1:** Clinical characteristics of rheumatoid arthritis patients.

Variable	Rheumatoid Arthritis n = 260
Age (years), mean ± SD	57 ± 12
Female sex, n (%)	247 (95)
Alcoholism, n (%)	40 (15.4)
Smoking, n (%)	28 (10.8)
Disease duration (years), mean ± SD	13.3 ± 9.3
BMI, mean ± SD	27.3 ± 5.4
DAS28-ESR score, mean ± SD	3.52 ± 1.3
Responders, n (%)	118 (45.4)
HAQ-DI score, mean ± SD	0.52 ± 0.53
Treatment	
csDMARDs, n (%)	260 (100)
Weekly methotrexate dose (mg), mean ± SD	13.2 ± 6.3
Monotherapy with 1 csDMARD, n (%)	86 (33.1)
Combined therapy with ≥2 csDMARDs, n (%)	174 (66.9)
bDMARDs, n (%)	34 (13.1)
Glucocorticoid, n (%)	133 (51.2)
Glucocorticoid dose (mg), mean ± SD	3.18 ± 3.9
Sulfasalazine, n (%)	104 (40.0)
Leflunomide, n (%)	44 (16.9)
Chloroquine, n (%)	53 (20.4)
Azathioprine, n (%)	11 (4.2)
Laboratory measurements	
Erythrocyte sedimentation rate (mm/h), mean ± SD	24.3 ± 11.2
Rheumatoid factor titers (UI/mL), mean ± SD	82.9 ± 236.8
CRP (mg/L), mean ± SD	10.9 ± 21.4
Serum AICAR levels (pg/mL), mean ± SD	276 ± 210
Serum IL-6 levels (pg/mL), mean ± SD	17 ± 28
Genetic characteristics	
rs2372536, *ATIC* gene	
Genotypes	
CC, n (%)	88 (34)
CG, n (%)	124 (48)
GG, n (%)	43 (18)
Allele, 2n = 520	
C, n (%)	300 (57)
G, n (%)	220 (43)
rs4673990, *ATIC* gene	
Genotypes	
GG, n (%)	85 (33)
AG, n (%)	128 (49)
AA, n (%)	47 (18)
Allele, 2n = 520	
G, n (%)	298 (57)
A, n (%)	222 (43)
rs4673993, *ATIC* gene	
Genotypes	
CC, n (%)	109 (42)
TC, n (%)	122 (47)
TT, n (%)	29 (11)
Allele, 2n = 520	
C, n (%)	340 (65)
T, n (%)	180 (35)

DAS28: disease activity for 28 joints; BMI: body mass index; HAQ-DI: Health Assessment Questionnaire Disability Index; ESR: erythrocyte sedimentation rate; CRP: C-reactive protein; AICAR, aminoimidazole carboxamidoribonucleotide; IL-6: Interleukin 6; qualitative variables are expressed in frequencies and quantitative variables in means ± standard deviations (SD).

**Table 2:** Comparison of characteristics in rheumatoid arthritis patients with response (DAS28 ≤ 3.2) vs. non-responders (DAS28 > 3.2).

Variables	Responders (DAS28 ≤ 3.2) n = 118	Non-Responders (DAS28 > 3.2) n = 142	*p*
Age (years), mean ± SD	57.4 ± 12	57.5 ± 12	0.87
Female sex, n (%)	112 (94)	135 (96)	0.54
Smoking, n (%)	8 (7.5)	20 (14.2)	0.09
Disease duration (years), mean ± SD	12.6 ± 8.8	13.8 ± 9.6	0.31
BMI, mean ± SD	27.2 ± 5.7	27.2 ± 5.1	0.85
HAQ-DI score, mean ± SD	0.32 ± 0.43	0.69 ± 0.56	<0.001
Treatment			
Weekly methotrexate dose (mg), mean ± SD	12.4 ± 3.76	14.0 ± 7.7	0.04
bDMARDs, n (%)	12 (10.2)	22 (15.6)	0.32
Glucocorticoid, n (%)	49 (41.5)	84 (59.6)	0.02
Glucocorticoid dose (mg), mean ± SD	2.48 ± 2.89	3.72 ± 4.53	<0.01
Sulfasalazine, n (%)	53 (44.9)	51 (36.2)	0.04
Leflunomide, n (%)	16 (13.6)	28 (20)	0.31
Chloroquine, n (%)	26 (22.0)	27 (19)	0.32
Azathioprine, n (%)	4 (3.4)	7 (5)	0.64
Laboratory measurements			
ESR (mm/h), mean ± SD	20.7 ± 9.4	27.3 ± 11.7	<0.001
Rheumatoid factor (UI/mL), mean ± SD	7 4.5 ± 179.8	86 ± 255	0.61
CRP (mg/L), mean ± SD	9.9 ± 23.2	11.7 ± 19.7	0.54
Serum AICAR levels (pg/mL), mean ± SD	284 ± 223	268 ± 200	0.51
Serum IL-6 levels (pg/mL), mean ± SD	12 ± 10	22 ± 36	0.002

DAS28: disease activity for 28 joints; BMI: body mass index; HAQ-DI: Health Assessment Questionnaire Disability Index; ESR: erythrocyte sedimentation rate; CRP: C-reactive protein; AICAR, aminoimidazole carboxamidoribonucleotide; IL-6: Interleukin 6; qualitative values are shown as frequencies and percentages; quantitative values are shown as medians and ranges. Comparison between groups of quantitative variables was performed using Student’s *t*-test. Comparison between groups of qualitative variables was performed using chi-square. The *p*-values were obtained by comparison between groups.

**Table 3:** Comparison of genotypic and allelic frequencies of polymorphisms rs2372536, rs4673990, and rs4673993 of *AICAR* gene among rheumatoid arthritis patients: responders (DAS28 ≤ 3.2) vs. non-responders (DAS28 > 3.2).

Variables n = 260	Responders (DAS28 ≤ 3.2) n = 118	Non-Responders (DAS28 > 3.2) n = 142	OR	95% CI	*p*
rs2372536, *ATIC* gene					
Genotypes					
CC, n (%)	43 (36)	45 (31.7)	-	-	
CG, n (%)	52 (44)	72 (50.7)	-	-	0.56
GG, n (%)	23 (20)	25 (17.6)	-	-	
CC vs. CG + GG	-	-	1.23	0.73–2.0	0.42
CG + CC vs. GG	-	-	0.88	0.47–1.65	0.69
Allele	2n = 236	2n = 284			
C, n (%)	138 (58)	162 (57)			0.10
G, n (%)	98 (42)	122 (43)			
rs4673990, *ATIC* gene					
Genotypes					
GG, n (%)	43 (36)	42 (29)	-	-	
AG, n (%)	53 (45)	75 (53)	-	-	0.41
AA, n (%)	22 (19)	25 (18)	-	-	
AA vs. AG + GG	-	-	0.91	0.48–1.73	0.78
AG + AA vs. GG	-	-	0.62	0.37–1.05	0.07
Allele					
G, n (%)	139 (59)	159 (56)	-	-	0.44
A, n (%)	97 (41)	125 (44)	-	-	
rs4673993, *ATIC* gene					
Genotypes					
CC, n (%)	47 (40)	62 (44)			
TC, n (%)	60 (51)	62 (44)			
TT, n (%)	11 (9)	18 (12)			
TT vs. TC + CC	-	-	0.70	0.32–1.56	0.39
TC + TT vs. CC	-	-	1.17	0.71–1.92	0.53
Allele	2n = 236	2n = 284			
C, n (%)	154 (65)	186 (65)	-	-	<0.05
T, n (%)	82 (35)	98 (35)	-	-	
Haplotypes					
Haplotype 1 CAC	47 (40)	54 (38)	0.89	0.43–1.86	0.08
Haplotype 2 GGT	38 (32)	42 (29)	0.86	0.40–1.84	0.14
Haplotype 3 CGC	18 (15)	23 (16)	-	-	-
Others	15 (13)	23 (16)	-	-	-

Qualitative values are shown as frequencies and percentages. CC/GG/CC: homozygous wild genotype. CG/AG/TC: heterozygous genotype. GG/AA/TT: homozygous polymorphic genotype. OR: odds ratio; 95% CI: 95% confidence interval. *p*-values were obtained by comparison between case and control groups.

**Table 4:** Associated factors with therapeutic non-response in RA patients.

	Univariate			Multivariate		
Variables	OR	95% CI	*p*	OR	95% CI	*p*
Female sex	1.18	0.23–6.09	0.83	-	-	-
Age (years)	0.99	0.96–1.02	0.64	-	-	-
Disease duration (years)	1.02	0.98–1.06	0.20	-	-	-
Smoking	2.19	0.66–1.29	0.19	-	-	-
HAQ-DI	3.94	1.97–7.87	<0.001	3.92	2.02–7.60	<0.001
Methotrexate, weekly dose	1.06	0.99–1.13	0.06	1.06	1.01–1.14	0.46
Glucocorticoid use	2.82	1.38–5.76	<0.001	2.87	1.44–5.72	<0.001
Sulfasalazine	0.48	0.48–0.24	0.04	0.49	0.25–0.95	0.03
ESR (mm/h)	1.08	1.04–1.13	<0.001	1.08	1.04–1.12	<0.001
IL-6 (pg/mL)	1.02	0.99–1.15	0.06	1.02	1.01–1.05	0.47
rs2372536	0.48	0.48–0.92	0.38	-	-	-
rs4673990	0.57	1.44–2.27	0.42	-	-	-
rs4673993	8.10	1.36–47.99	0.02	4.50	1.04–19.34	0.04

Multivariable logistic regression analysis. Dependent variable therapeutic response, OR: odds ratio; 95% CI: 95% confidence interval. Crude ORs were obtained using the Intro method. Adjusted ORs were obtained using the backward stepwise method. HAQ-DI: Health Assessment Questionnaire Disability Index; ESR: erythrocyte sedimentation rate; IL-6: Interleukin 6.

**Table 5:** Sub-analysis of rheumatoid arthritis patients on methotrexate monotherapy comparing responders (DAS28 ≤ 3.2) vs. non-responders (DAS28 > 3.2).

Variables	Responders (DAS28 ≤ 3.2) n = 35	Non-Responders (DAS28 > 3.2) n = 51	*p*
Age (years), mean ± SD	57.4 ± 12	61 ± 12	0.24
Female sex, n (%)	35 (100)	50 (98)	0.40
Smoking, n (%)	1 (4.2)	6 (12)	0.28
Disease duration (years), mean ± SD	13 ± 10	13 ± 8	0.91
BMI, mean ± SD	27 ± 5	26 ± 5	0.84
HAQ-DI score, mean ± SD	0.20 ± 0.42	0.61 ± 0.55	<0.01
Treatment			
Weekly methotrexate dose (mg), mean ± SD	11.5 ± 3	14.3 ± 9	0.06
Laboratory measurements			
ESR (mm/h), mean ± SD	21.5 ± 9	28.2 ± 13	0.01
Rheumatoid factor (UI/mL), mean ± SD	102 ± 187	111 ± 401	0.88
CRP (mg/L), mean ± SD	8.3 ± 11.1	9.5 ± 16	0.69
Serum AICAR levels (pg/mL), mean ± SD	185 ± 156	255 ± 160	0.04
Serum IL-6 levels (pg/mL), mean ± SD	10 ± 9	18 ± 25	0.06
rs2372536, n (%)	CC 12 (36) CG 16 (49) GG 5 (15)	CC 19 (40) CG 22 (47) GG 6 (13)	0.32
rs4673990, n (%)	CG 15 (43) AG 13 (37) AA 7 (20)	CG 15 (31) AG 26 (54) AA 7 (15)	0.69
rs4673993, n (%)	CC 15 (43) TC 15 (43) TT 5 (14)	CC 23 (48) TC 20 (42) TT 5 (10)	0.42

DAS28: disease activity for 28 joints; BMI: body mass index; HAQ-DI: Health Assessment Questionnaire Disability Index; ESR: erythrocyte sedimentation rate; CRP: C-reactive protein; AICAR, aminoimidazole carboxamide ribonucleotide; IL-6: Interleukin 6. Qualitative values are shown as frequencies and percentages; quantitative values are shown as medians and ranges. Comparison between groups of quantitative variables was performed using Student’s *t*-test. Comparison between groups of qualitative variables was performed using chi-square. The *p*-values were obtained by comparison between groups.

## Data Availability

The database used to support the findings of this study is available on request. If this database is required, please direct the correspondence to Ana M. Saldaña-Cruz (ana.saldanac@academicos.udg.mx).
